# Magnetic resonance imaging of breast augmentation: a pictorial review

**DOI:** 10.1007/s13244-016-0482-9

**Published:** 2016-03-09

**Authors:** Ting Wong, Lai Wan Lo, Po Yan Eliza Fung, Hiu Yan Miranda Lai, Hoi Lam Helen She, Wing Kei Carol Ng, King Ming Kimmy Kwok, Chiu Man Lee

**Affiliations:** Department of Radiology, Block B, LG1, Princess Margaret Hospital, 2-10 Princess Margaret Hospital Road, Lai Chi Kok, Kowloon, Hong Kong; Department of Diagnostic and Interventional Radiology, E1, Kwong Wah Hospital, 25 Waterloo Road, Yau Ma Tei, Kowloon, Hong Kong

**Keywords:** MRI, Breast, Augmentation, Implant, Malignancy

## Abstract

**Abstract:**

The increasing prevalence of breast augmentation presents new challenges in breast imaging interpretation. Magnetic resonance imaging (MRI) is recognized as the gold standard for the evaluation of augmented breasts. This article reviews the MRI features of different breast augmentation techniques, their associated complications, and the role of MRI in the assessment of concurrent breast abnormalities.

***Teaching Points*:**

• *MRI has the highest sensitivity and specificity for implant rupture detection*.

• *MRI is able to discriminate the nature of implanted prosthesis or injected materials*.

• *Sensitivity of cancer detection by MRI is not reduced through implants*.

## Introduction

Breast augmentation or mammoplasty has become increasingly prevalent in recent years for aesthetic reasons and for reconstruction in breast cancer patients. Clinical diagnosis for augmentation-related complications is notoriously difficult, as the physical examination findings are usually non-specific [1]. Therefore, breast imaging has an essential role in investigating breast complaints in this patient group. Because of the wide range of surgical techniques and materials employed in mammoplasty, it is a new challenge for radiologists to interpret images of augmented breasts. Although mammogram and ultrasound are the modalities of choice for initial work-up, MRI is regarded as the gold standard, particularly in evaluating the integrity of prosthetic implants [2].

MRI has preeminent strengths over other imaging modalities despite its higher cost. MRI has high sensitivity and specificity in depicting subtle abnormalities [3]. Using different pulse sequences, MRI can differentiate amongst water, fat, muscle, and implant materials with high spatial and soft tissue resolution [4]. This is helpful in pre-surgical planning such as in implant removal, where MRI can depict the presence and extent of implant-related complications. It is also useful in assessing known or suspected malignancy. Its lack of ionizing radiation is another advantage.

In this article, we describe the MRI features of different breast augmentation and reconstruction techniques, their associated complications and the role of MRI in the evaluation of concurrent breast pathologies.

### Breast prosthesis

**Fig. 1:**
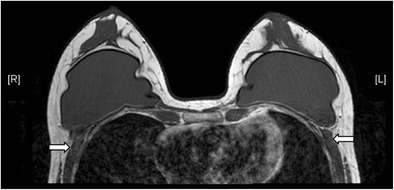
Retroglandular position of implants. T1-weighted axial MR image shows the retroglandular position of bilateral silicone gel-filled implants, which are entirely anterior to the pectoral muscles (arrows)

**Fig. 2:**
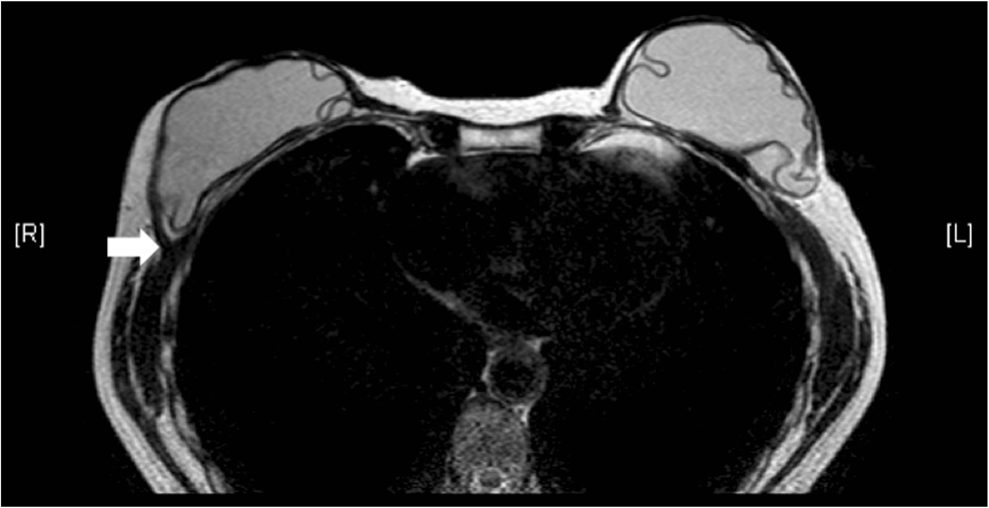
Retropectoral position of implants. T2-weighted MR image on axial plane demonstrates the retropectoral position of the right silicone gel-filled implant. Note the right pectoral muscle (arrow) is split by the implant

Numerous styles and types of breast implants have been used since it was first invented in 1962 [5]. The commonly used prosthetic breast implants nowadays can be categorized by the number of lumens and their filling materials. A single-lumen implant is a multilayered shell filled with silicone gel or, less commonly, saline solution. A standard double-lumen implant is filled with silicone gel in the inner lumen and with saline solution in the smaller outer lumen. An inverse double-lumen implant is filled with saline solution in the inner lumen, which can be expanded as necessary, and with silicone gel in the outer lumen. The implant can be placed in a subglandular (retroglandular) location, which is anterior to pectoralis major muscle (Fig. 1) or in a subpectoral (retropectoral) location (Fig. 2) [6].

**Fig. 3:**
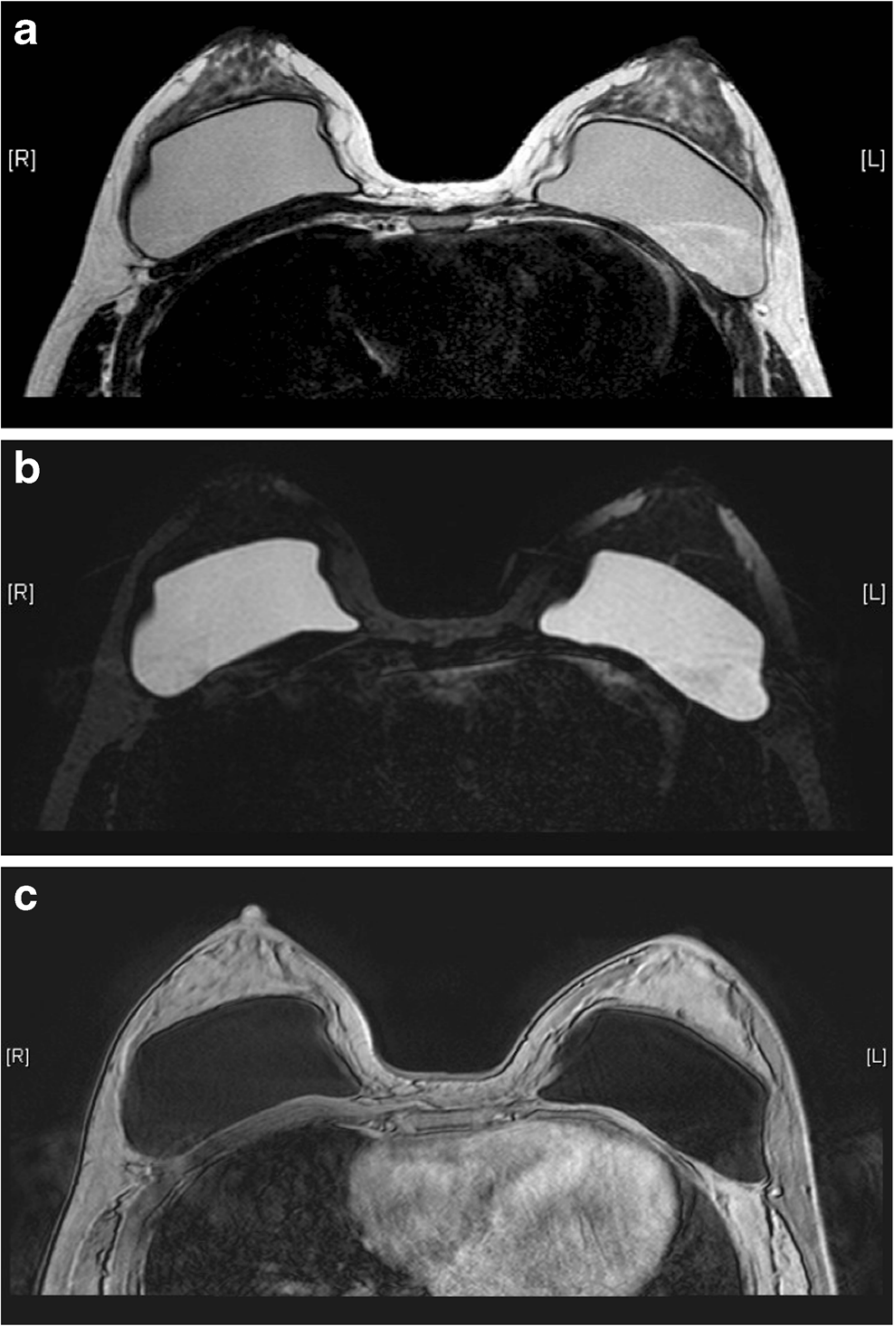
Silicone gel-filled implants. T2-weighted (**a**), silicone-only (**b**), and silicone-saturated (**c**) axial MR images show the specific signal intensities of silicone, thus rendering the differentiation between silicone gel-filled and saline-filled implants possible

**Fig. 4:**
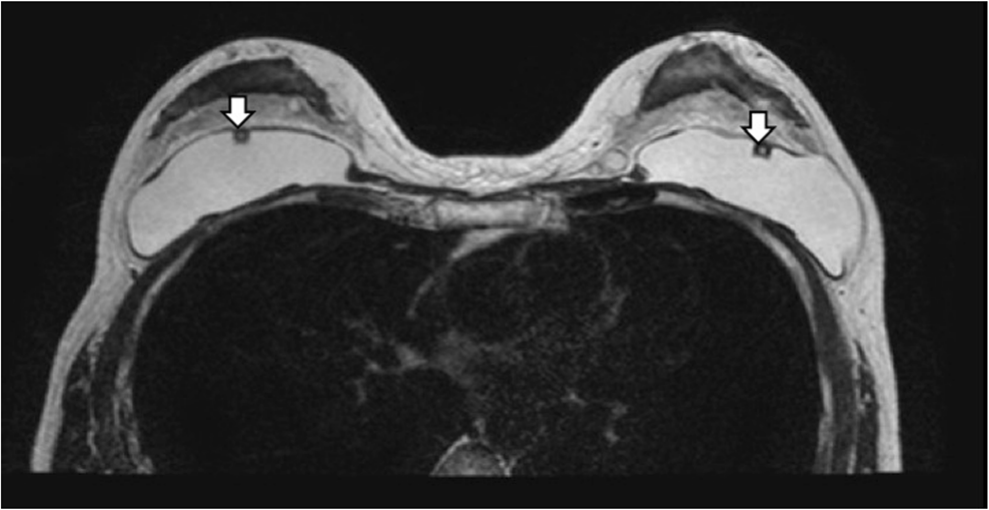
Saline-filled implants. High signal intensity identical to water is seen in saline-filled implants on T2-weighted sequence. Note the valves (arrows) at the anterior aspect of the shells, which are characteristic of saline-filled implants, but not silicone gel-filled implants

Useful MRI sequences to investigate prosthetic implants include fast T2-weighted sequence, silicone-only sequence (silicone hyperintense, water hypointense), and silicone-saturated sequence (silicone hypointense, water hyperintense). Saline and silicone give different signal intensities depending on the pulse sequences, enabling their differentiation and localization (Figs. 3 and 4).

## Normal appearance

**Fig. 5:**
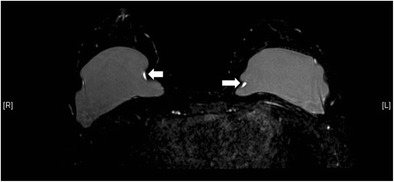
Periprosthetic fluid. T2-weighted fat-saturated axial MR image shows trace amount of periprosthetic fluid bilaterally (arrows), which is a common normal finding

**Fig. 6:**
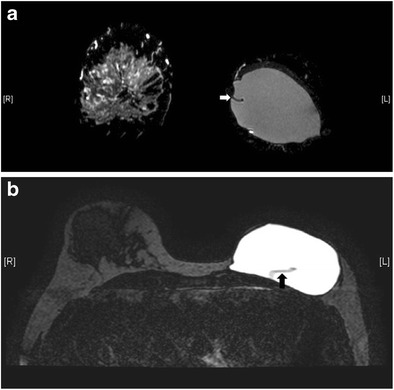
Radial folds. T2-weighted fat-saturated coronal (**a**) and silicone-only axial (**b**) MR images of the same patient with silicone gel-filled implants show a hypointense line running from the periphery, which is perpendicular to the normal intact left silicone gel-filled implant shell, suggestive of a radial fold (arrow)

In a normal implant, the shell is intact with a surrounding thin, fibrous capsule. It is common to find a small to moderate amount of peri-prosthetic fluid, which is reactive in nature (Fig. 5). The presence of radial folds is normal. They are perpendicular infoldings of the shell into the silicone gel or saline solution, extending inwards from the periphery (Fig. 6). This can be mistaken as implant rupture by the inexperienced eye.

## Complications

Early postoperative complications include seroma, hematoma, and infection [6]. Seroma is a fluid collection in the surgical bed, usually adjacent to the scar. A seroma may form an abscess if infected, which would show rim-enhancement on MRI. MRI can determine the age of a hematoma, with acute-subacute hematoma appearing hyperintense on the T1 sequence.

## Late postoperative complications

1. Capsular contracture
  The thin, fibrous capsule surrounding the implant, as previously mentioned, is a normal finding. The capsule should be elastic and clinically impalpable. Excessive scarring will result in capsular contracture, which is one of the most common complications of prosthetic implants [3]. It can occur anytime post-operatively, but usually within the first few months. This condition is best diagnosed clinically [7], with the patient complaining of pain and breast disfigurement. On examination, the thick, fibrous tissue may become fixed and palpable. Although not always present, possible imaging findings include alteration of implant contour, which becomes asymmetrical, irregular, and more spherical in shape, with infoldings or tenting. Coarse peri-implant calcification and peripheral enhancement may also be seen [3, 6] (Fig. 7).

**Fig. 7:**
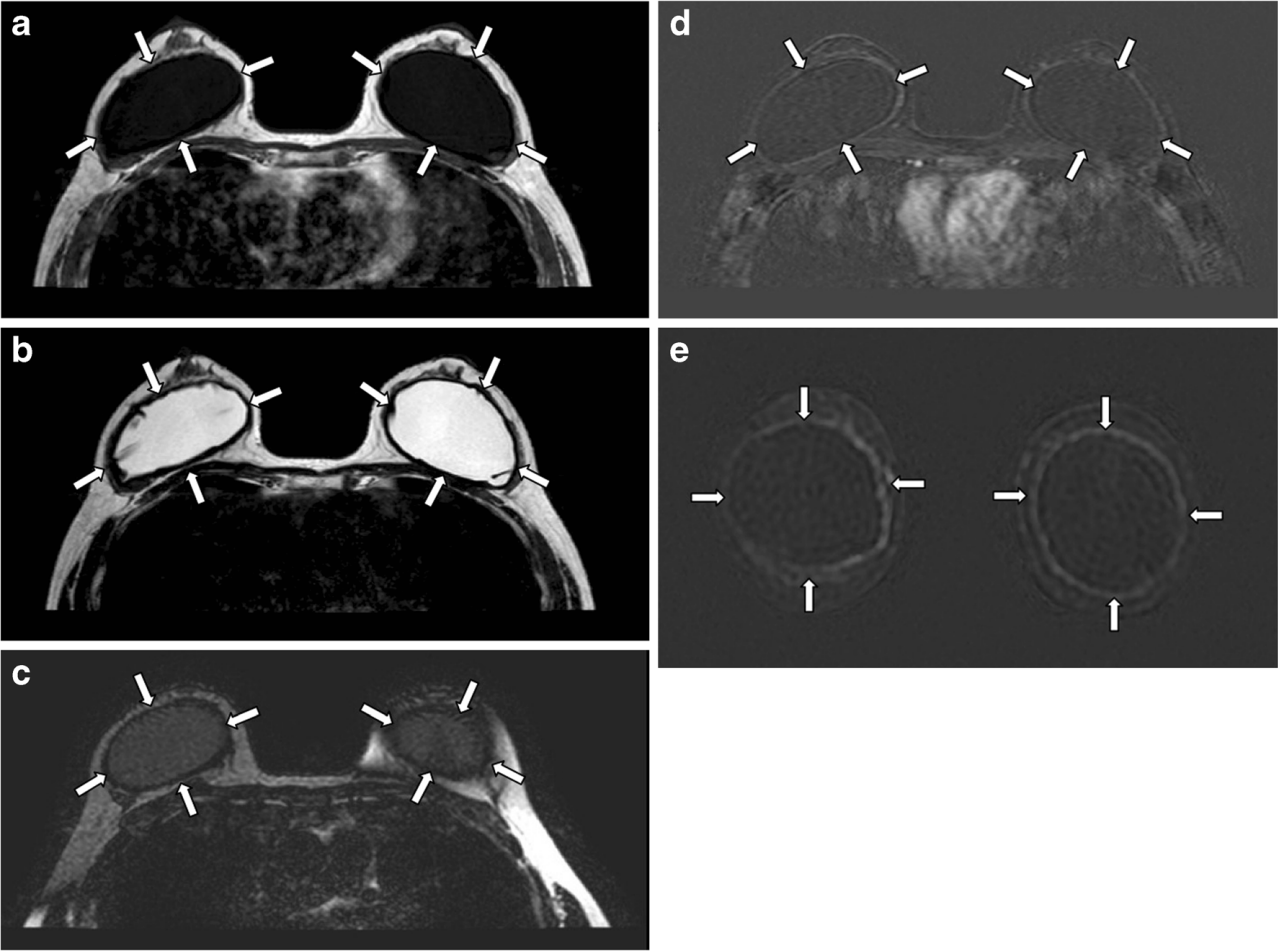
Capsular contracture. Axial MR images of T1-weighted (**a**), T2-weighted (**b**), and silicone-only (**c**) sequences show thick low signal fibrous capsules surrounding both implant shells (arrows). Note that the implant material signal is low on silicone-only sequence (**c**), which suggests that these are saline-filled implants. T1-weighted post contrast images with subtraction on axial (**d**) and coronal (**e**) plane show enhancement of the pseudocapsules
2. Bulging or herniation
  This describes protrusion of the implant shell through a focal weakened or torn part of the fibrous capsule. It is called the “rat-tail sign” if very pronounced. This can potentially lead to rupture [2, 3] (Fig. 8)

**Fig. 8:**
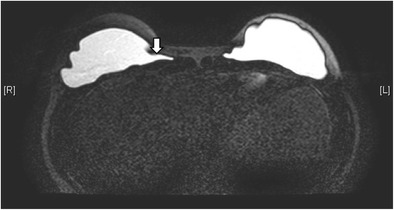
Rat tail sign. T2-weighted, fat-saturated axial MR image shows protrusion of the implant shell through a focally weakened part of the fibrous capsule at medial aspect of right breast, simulating a rat tail (arrow)
3. Implant rupture
  Implant rupture is one of the key complications of breast implants. Its risk increases with implant age and is more common for retropectoral than retroglandular implants [8]. They can be classified as intracapsular or extracapsular rupture, depending on where the ruptured implant material is located with respect to the fibrous capsule. Intracapsular implant rupture is more common, with the leaked silicone gel or saline solution confined within the fibrous capsule; whereas for extracapsular implant rupture, the leaked material is located outside of the fibrous capsule. Rupture can also be classified according to the degree of implant shell collapse as uncollapsed, minimally collapsed, partially collapsed, and fully collapsed.
  MRI has the highest sensitivity and specificity for implant rupture detection compared with ultrasound and mammogram. The sensitivity is between 80 % and 90 %, and the specificity is between 90 % and 97 % [3]. There are various MRI signs which signify uncollapsed intracapsular rupture, where small amount of silicone or saline can be found outside of the shell.
  “Subcapsular line sign”. A hypointense line is noted running almost parallel to and just beneath the fibrous capsule due to a thin layer of implant material between the shell and the fibrous capsule [2, 3] (Fig. 9).

**Fig. 9:**
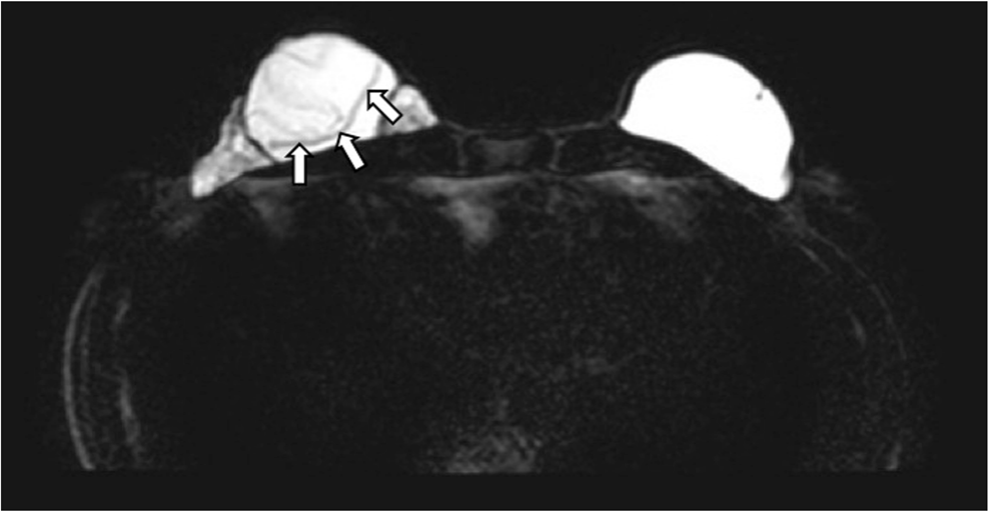
Subcapsular line sign. Silicone-only axial MR image shows a hypointense line (arrow) running almost parallel to and just beneath the fibrous capsule in right breast, due to a thin layer of silicone between the shell and the fibrous capsule
  “Pull away”, “open loop”, or “undercapsular streaks” sign. This refers to the presence of hypointense lines, which are characteristically parallel to the capsule, due to localized leakage of implant material. This causes a small displacement of the capsule [2].
  “Keyhole”, “noose”, or “inverted-loop” sign. It appears when there is further progression of the “pull away sign”. It is the leakage of implant material into the invaginated implant shell, with the two membranes not touching each other [2, 3] (Fig. 10).

**Fig. 10:**
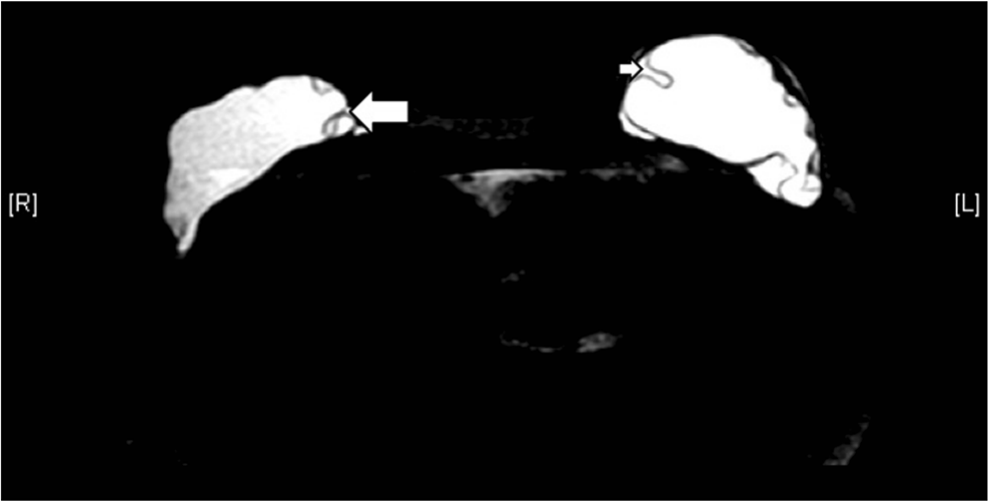
Keyhole sign (left breast) and teardrop sign (right breast). Silicone-only axial MR image shows keyhole sign (small arrow) at left breast. The invaginations of implant shell do not touch each other. Tear-drop sign is seen at right breast (large arrow), with the invaginated membranes contacting one another
  “Tear-drop sign” differs from “keyhole sign” in that the invaginated membranes contact one another (Fig. 10).
  “Linguine sign” is the presence of curvilinear hypointense lines, which represents the collapsed shell, floating in the high-signal intensity silicone gel [3] (Fig. 11). This sign is only present in collapsed intracapsular rupture.

**Fig. 11:**
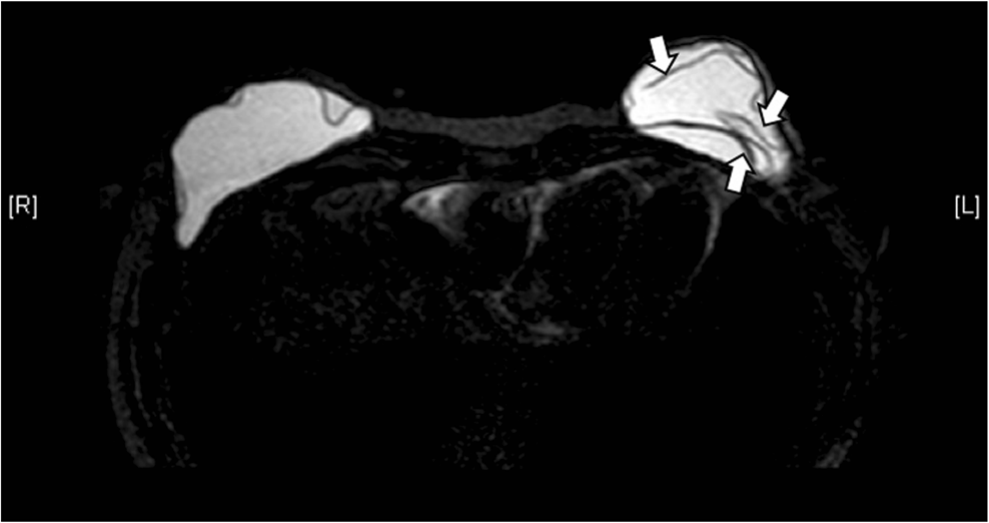
Linguine sign. Silicone-only axial MR image shows curvilinear hypointense lines (arrows) in the left breast implant, compatible with the collapsed shell floating in the high-signal silicone gel. This is called the linguine sign
  “Salad oil sign” or “droplet sign” occurs when there are small, hyperintense foci within the silicone gel on T2-weighted images, or hypointense foci on water-suppressed images. This is due to the presence of saline drops in silicone gel when there is an intracapsular rupture of a double lumen implant. The sign can be seen in both uncollapsed and collapsed intracapsular rupture. The presence of this sign in a single lumen silicone implant means there is water droplet in the silicone gel. Without other evidence, it is not a reliable sign of implant rupture, but its presence should prompt the search of other signs of intracapsular rupture [3].
  Extracapsular rupture means the leakage of the implant material beyond the fibrous capsule into the surrounding tissues. It happens when both the implant shell and the fibrous capsule are ruptured (Figs. 12 and 13). Free silicone can be seen as discrete foci, usually with identical signal intensity as the silicone gel inside the implant (Fig. 14). They appear isointense to hypointense on T1-weighted, fat-suppressed images and is of high signal intensity on water-suppressed T2-weighted images [6]. With time, silicone granuloma formation may occur, showing enhancement that may mimic breast carcinoma. Differentiation solely on the basis of imaging can be difficult. Therefore, pathological diagnosis by biopsy is warranted if there is suspicion [6]. In the case of saline-filled implant extracapsular rupture, free hydrosaline solution is usually resorbed by the body, with the remaining collapsed implant shell being the only imaging clue (Fig. 15).

**Fig. 12:**
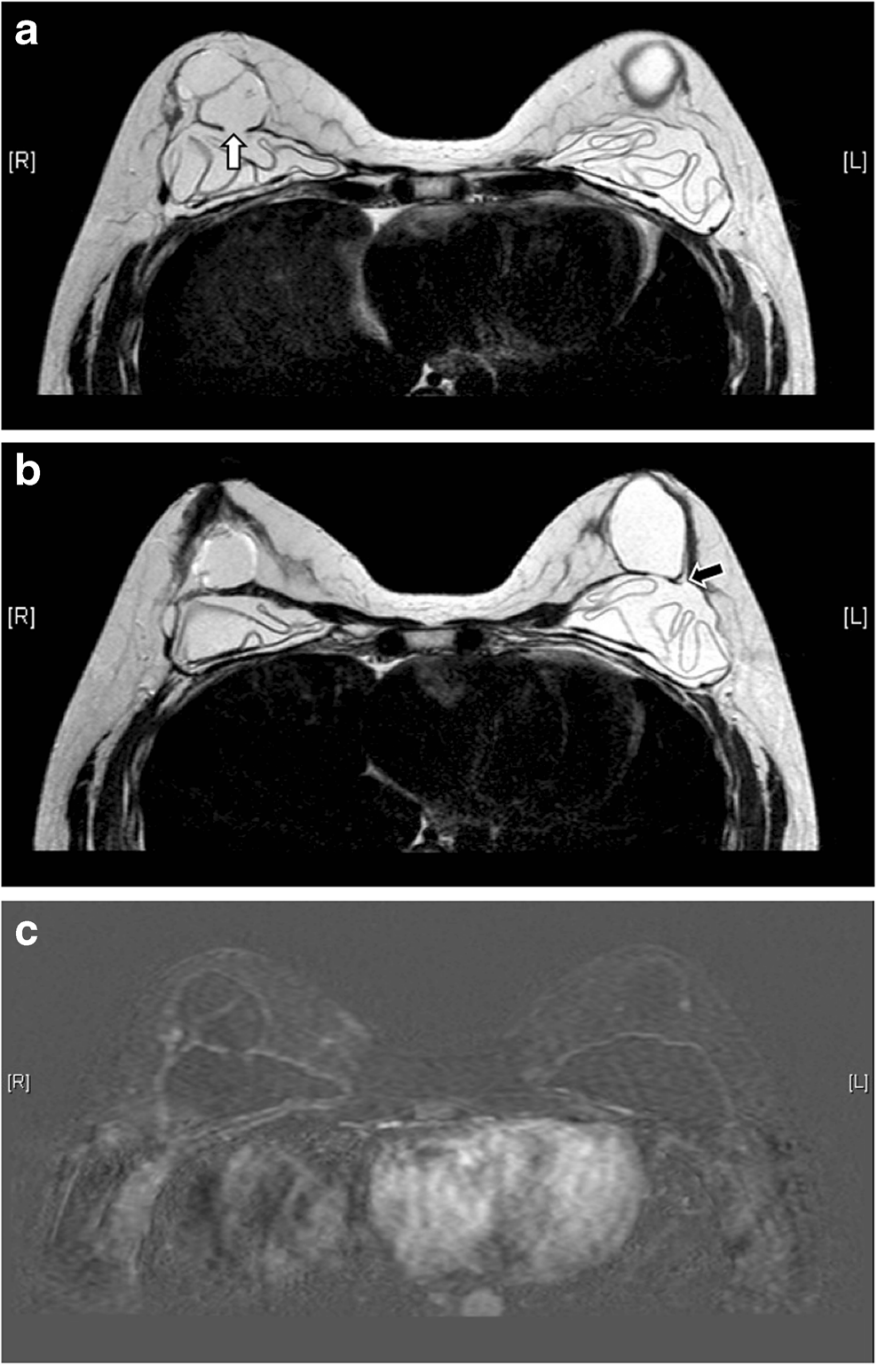
Extracapsular rupture. T2-weighted axial MR images (**a**, **b**) show bilateral extracapsular ruptures. Defects are seen at bilateral fibrous capsules (white arrow on the right and black arrow on the left). Both collapsed implants demonstrate the linguine sign. On the T1-weighted post-contrast image with subtraction (**c**) there is a thin reactive rim of enhancement surrounding the collapsed implants

**Fig. 13:**
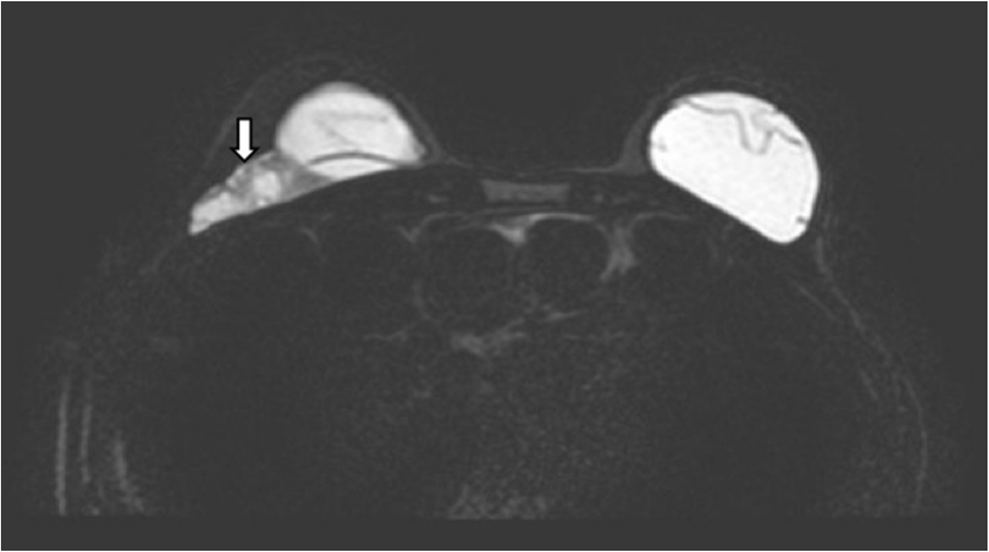
Extracapsular rupture of the right silicone gel-filled implant. Silicone-only axial MR image shows extracapsular rupture of the right silicone gel-filled implant (arrow)

**Fig. 14:**
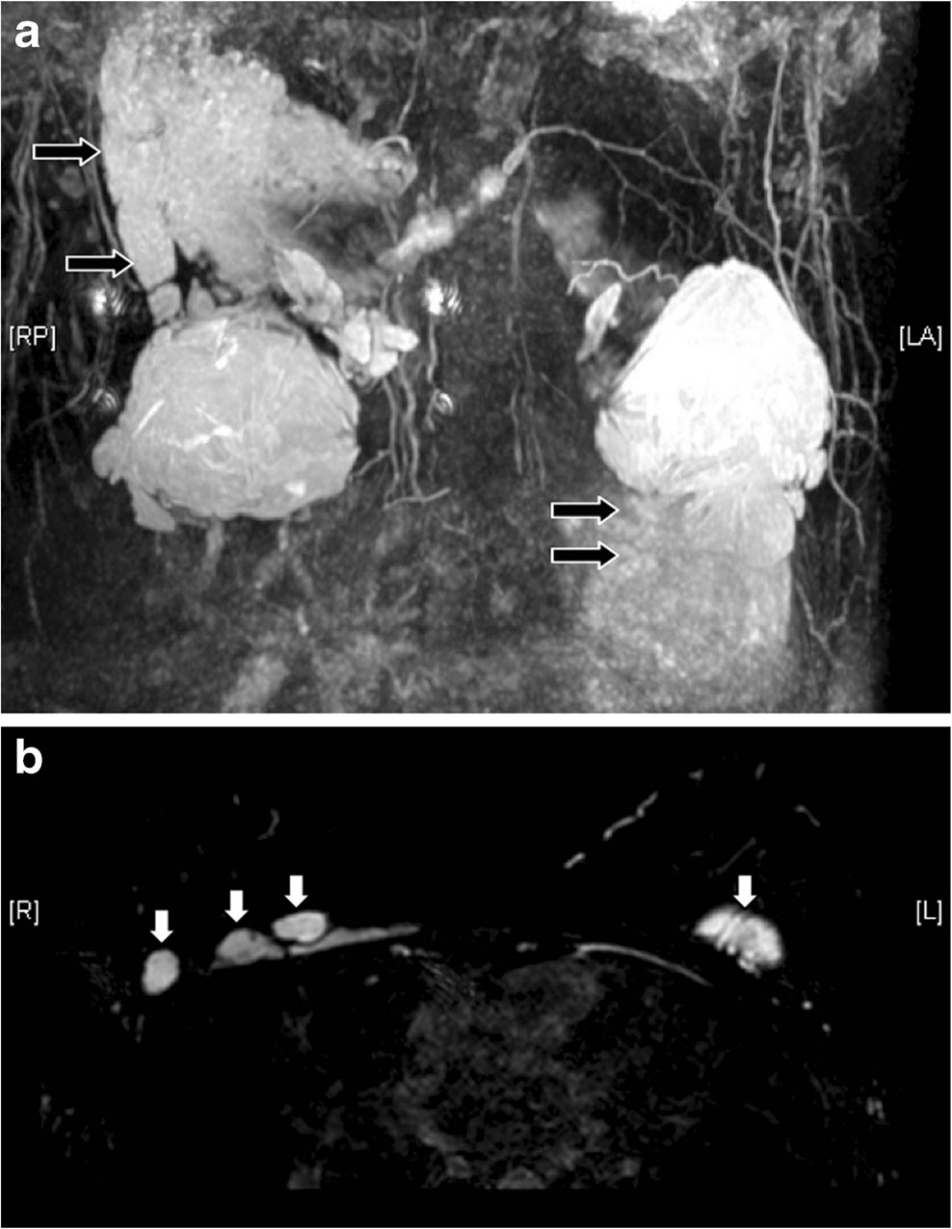
Free silicone at chest wall. Reconstructed T2 coronal MR image with MIP (**a**) reveals extensive silicone leakage (black arrows) from bilateral silicone gel-filled implants. The free silicone has identical signal with the silicone gel inside the implants. On T2-weighted, fat-saturated axial image (**b**) discrete free silicone foci are seen over the chest wall (white arrows)

**Fig. 15:**
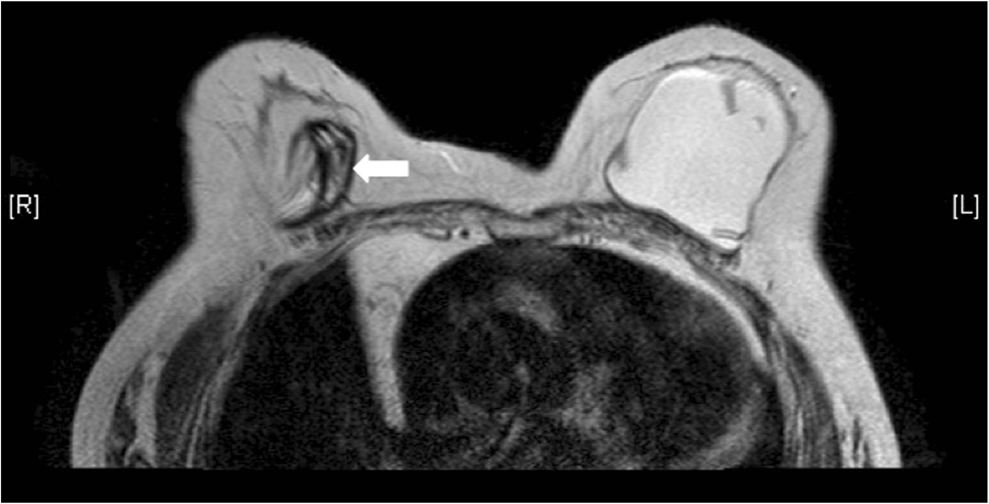
Collapsed right saline-filled implant. T2-weighted axial MR image shows collapsed right implant shell (arrow). The leaked hydrosaline solution has been resorbed
4. Gel bleed
  This is actually a misnomer. It describes the penetration of silicone fluid (rather than the gel) through the membrane micropores of an intact implant shell. The fluid can migrate to the other parts of the body, such as the regional lymph nodes, once it is separated from the outer shell. MRI cannot detect the normal transudation of microscopic amount of fluid, unless it is extensive and forms an inverted teardrop sign [3]. The phenomenon of gel bleed is improved after the invention of new cohesive gel implants [2]

### Polyacrylamide gel (PAAG) injection

Injectable breast augmentation using PAAG has been popular in China, Eastern Europe, and South America since 1997 [9]. PAAG contains 95-97.5 % of water [9], thus has similar signal intensities to that of water on MRI and is best depicted on T2-weighted sequence. Sometimes patients might have unknown or more than one type of material injected to their breasts. Silicone-only and silicone-saturated sequences would be of great help in differentiating PAAG from silicone.

Turbo spin-echo, T2-weighted, non-fat suppressing sequence is the best sequence to detect the location or extent of PAAG [9]. PAAG is of high signal intensity, while fat and glandular tissue have a signal intensity of light gray and dark gray, respectively. Pectoral muscle is hypointense on this sequence. Dynamic contrast-enhanced T1 study is also useful to assess the complications of PAAG injection such as inflammatory reaction and abscess [9].

**Fig. 16:**
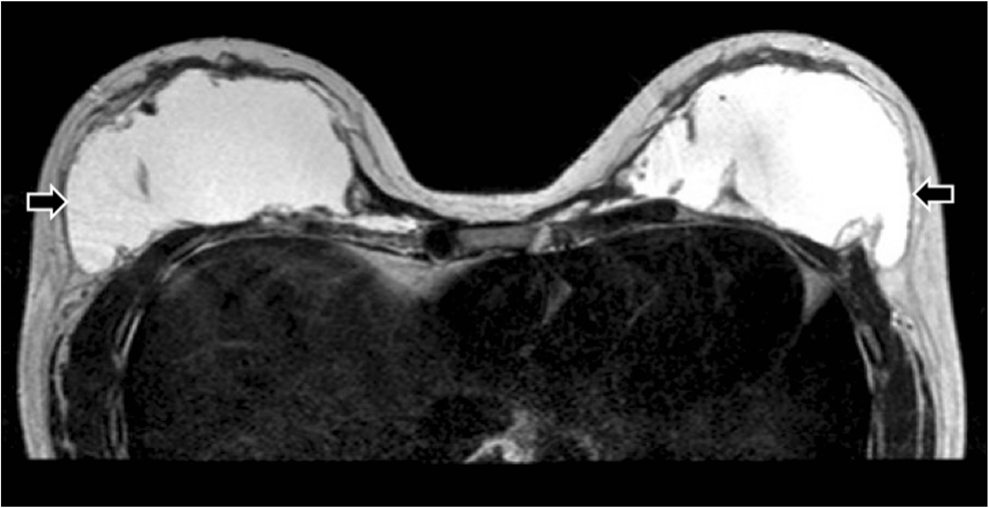
Desirable retroglandular position of PAAG. T2-weighted axial MR image demonstrates uncomplicated PAAG injection, with a collection of homogeneous T2 hyperintense material (arrows) at the retroglandular region of each breast, located anterior to the pectoral muscles

**Fig. 17:**
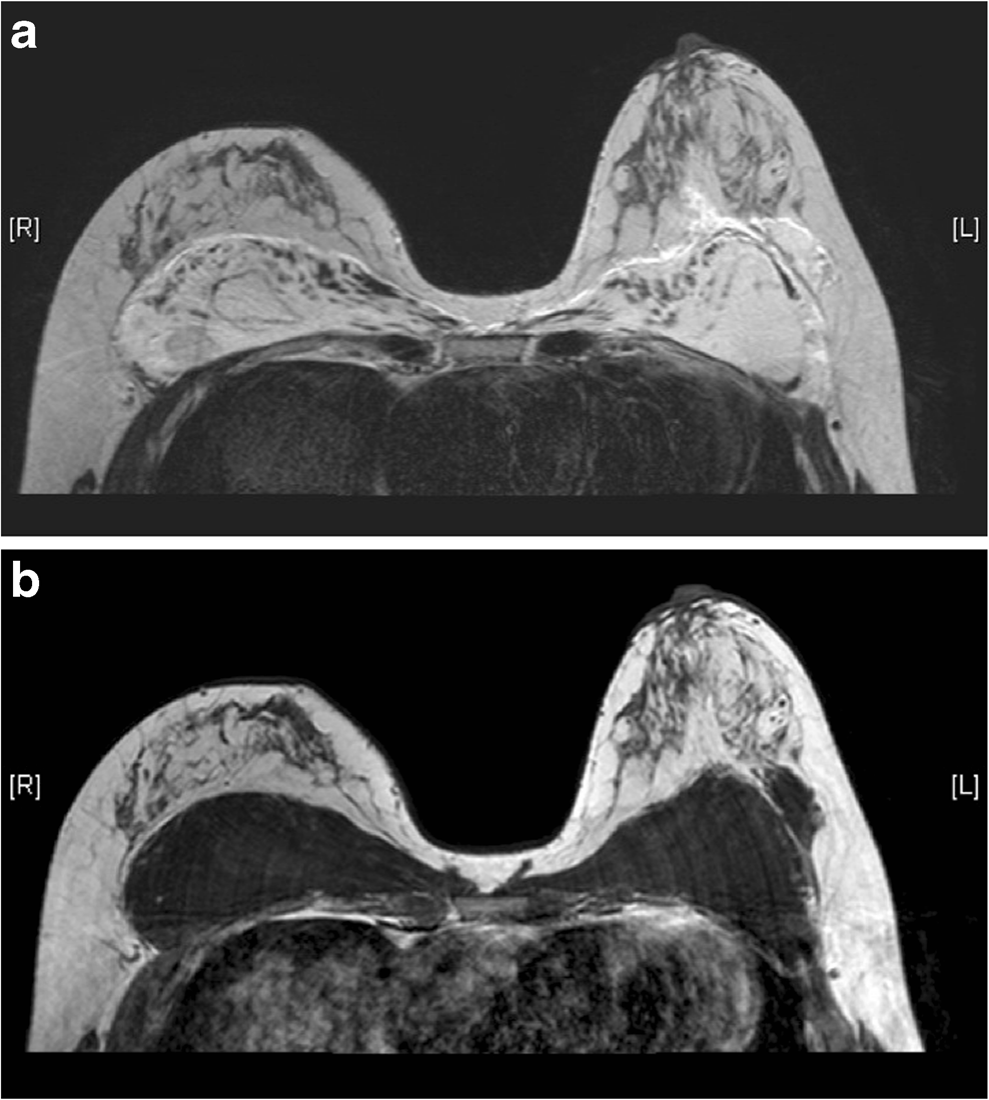
Asymmetric breasts after PAAG injection. T2-weighted (**a**) and T1-weighted (**b**) axial MR images show the undesirable outcome of breast asymmetry after augmentation by PAAG injection

For uncomplicated injections, PAAG should appear as a large collection of homogeneous T2 hyperintense signal in the retroglandular region, anterior to the pectoral muscles [9] (Fig. 16). PAAG does not induce as much physiological response to foreign body as other injectable augmentation materials. It, therefore, tends to lack a thick surrounding fibrous capsule. It was reported that injected gel were unable to form a single blob in 81.5 % of augmented breasts [9]. As the injection procedure is performed blindly without image guidance, there is a high risk of gel migration if the gel is undesirably injected outside of the retroglandular space. This can potentially lead to breast asymmetry related to gel migration or due to the difference in the amount of gel injected (Fig. 17). Breast asymmetry was found in 20-52.9 % in previous studies [9, 10].

**Fig. 18:**
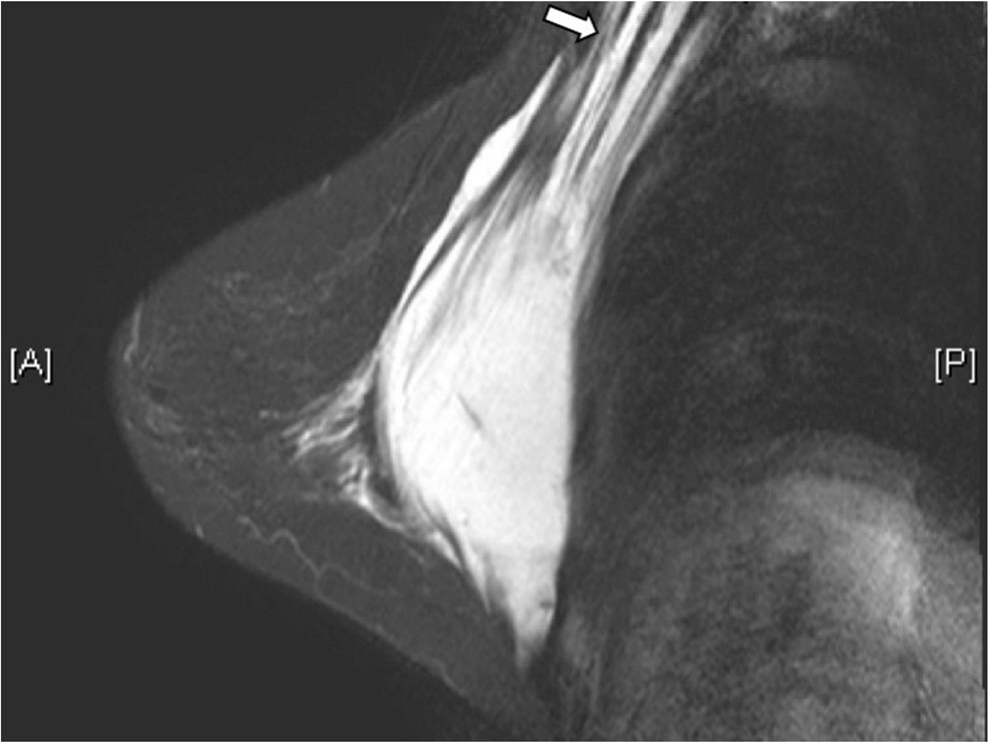
Intrapectoral migration of PAAG. T2-weighted, fat-saturated sagittal MR image reveals PAAG tracking along fascial planes of the pectoral muscle (arrow)

**Fig. 19:**
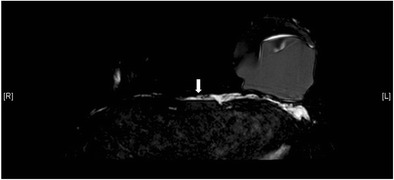
Chest wall extension of PAAG in a patient with history of left breast augmentation by PAAG injection and silicone gel-filled implant. Extension of PAAG to chest wall is clearly shown on this T2-weighted, fat-saturated MR axial image. Silicone is of low signal while PAAG is of high signal on this sequence. Medial extension of PAAG to pre-sternal region is noted (arrow)

**Fig. 20:**
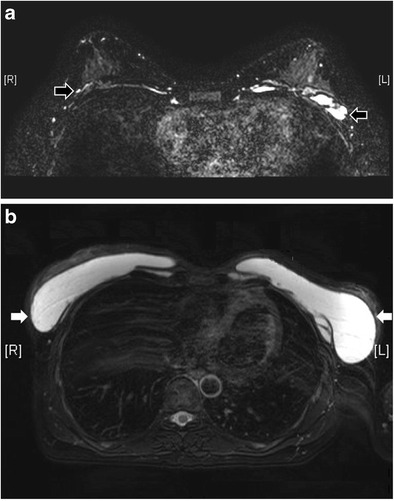
PAAG at anterior abdominal wall. Axial T2-weighted fat-suppressed image show there is minimal PAAG in bilateral retroglandular spaces (black arrows) (**a**). Instead, the main collections are found over anterior abdominal wall (white arrows) (**b**)

Outside the retroglandular space, PAAG tends not to coalesce but forms multiple small loculations. In the intrapectoral region, the gel can track along musculofacial planes (Fig. 18). In the retropectoral region, it may extend into the intrathoracic or extra pleural space. It can form nodules subcutaneously, particularly at the common sites of injection, namely the inframammary crease or axillary region. Sometimes it can migrate medially to the pre-sternal region (Fig. 19) and up to shoulder and infraclavicular region. Nodal involvement is not uncommon. Extension to the abdominal wall (Fig. 20) is sometimes encountered. If injected to the intraglandular region, PAAG can cause glandular atrophy and skin necrosis as well [11]. Such propensity of gel migration often leads to incomplete surgical removal. Distant gel migration was detected in 8.9 % of patients after PAAG injection [10].

**Fig. 21:**
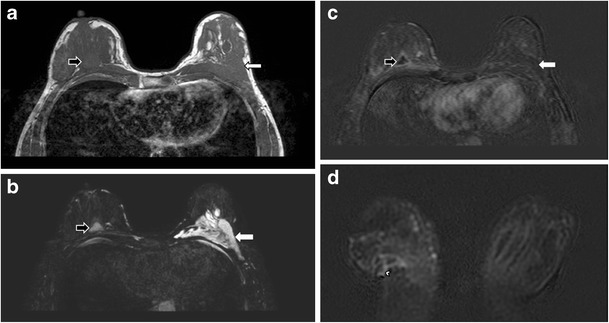
PAAG injection complicated with recurrent right breast abscess. Axial MR images of T1-weighted (**a**) and T2-weighted, fat-saturated (**b**) sequences depict a T1 hypointense and T2 hyperintense collection (black arrows) at the right breast. It shows rim enhancement after contrast injection (**c**). A sinus tract (arrowhead) is demonstrated at the lower outer aspect of the collection on the T1-weighted post-contrast coronal image with subtraction (**d**). Note the T1 low signal (**a**), T2 high signal (**b**) PAAG collection without any enhancement at the left retroglandular region (white arrows) (**c**)

Infection and abscess formation are common complications, especially if the procedure is performed without sterile technique. Of PAAG augmentation mammoplasty, 14.7 % was reported to be complicated with infection [9]. In the acute phase, the augmented breast is enlarged, with internal low-signal intensity foci inside the gel on T2-weighted MRI images, signifying pus formation. Contrast-enhanced MRI shows a thick nodular and irregular rim of contrast enhancement, which is helpful in delineating the extent of infection. For chronic infection, sinus formation may be seen (Fig. 21).

### Liquid silicone injection

**Fig. 22:**
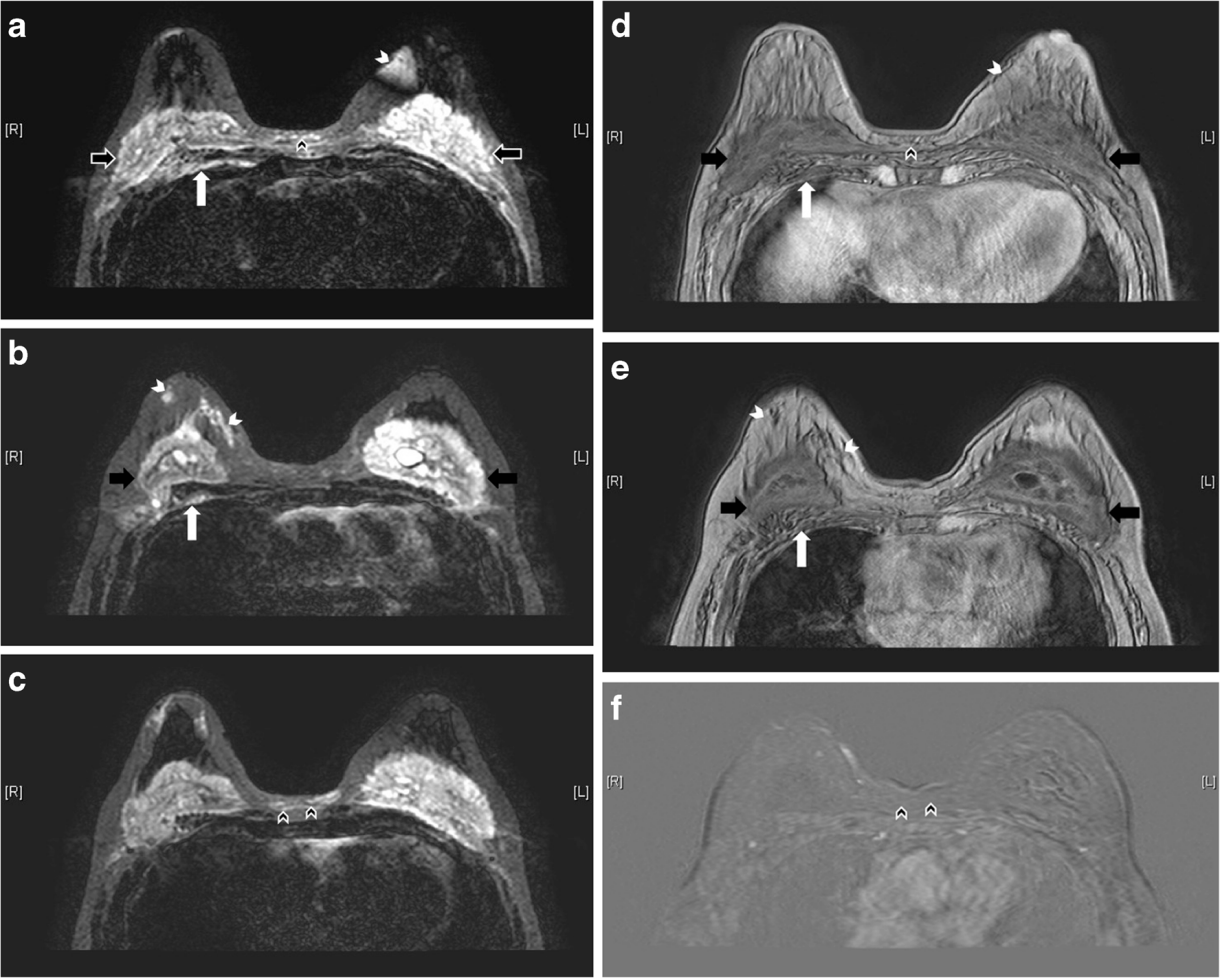
Free silicone injection. High signal collections (black arrows) are noted at the retroglandular regions of both breasts on these axial MR images of silicone-only sequence (**a**–**c**), compatible with free silicone. Collections are also seen in premammary fat (white arrowheads), retropectoral region (white arrows), and pre-sternal region (black arrowheads). They are suppressed on silicone-saturated sequence (**d**, **e**). No enhancement is noted over the pre-sternal collection (black arrowheads) on T1-weighted post-contrast image with subtraction (**f**)

This augmentation technique was introduced in 1940s and is now banned in view of its adverse effects. The free silicone is injected into the breast parenchyma, pectoralis muscles, or both (Fig. 22). Complications of liquid silicone injection include skin necrosis, silicone migration, embolism, infection, lymphadenopathy, and granuloma formation. Pathologically, silicone granulomas are nodules that contain small silicone parts associated with abundant fibrous reaction [12]. Their typical imaging appearance is that of a spherical nodule with peripheral calcifications. They can occasionally mimic malignancy, with spiculated borders [6]. Their signal intensity on MRI is related to the impurities in the silicone preparation [12]. Sometimes there may be mixed signals for injected silicone due to the formation of fibrous tissue, which results in low signal, or the presence of silicone granulomas, which decrease the signal intensity on T2-weighted sequence.

### Paraffin injection

**Fig. 23:**
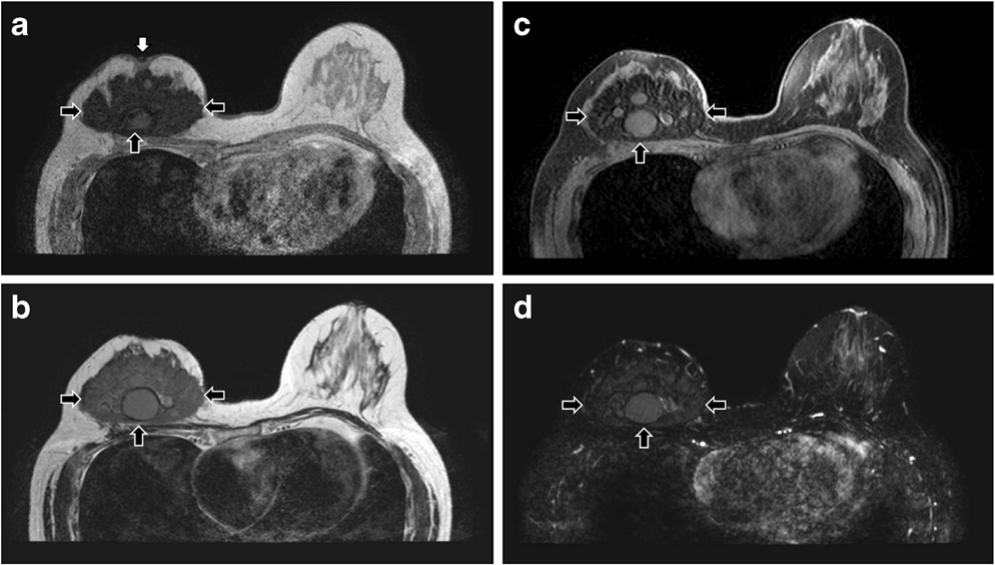
Unilateral right breast paraffin injection. T1-weighted (**a**), T2-weighted (**b**), T2-weighted, fat-saturated (**c**), and T1-weighted, fat-saturated, post-contrast (**d**) axial MR images show a right breast mass (black arrows) with T1 and T2 low to intermediate signals and minimal contrast enhancement. Nipple retraction (white arrow) is observed (**a**)

It was once a popular breast augmentation method in the early 1900s, but it is abandoned nowadays owing to serious complications. Paraffinoma formation is common [6]. Its MRI signals and fat-saturated features vary with the purity of the injected materials and depend on whether the paraffin is in liquid or semi-solid state, which is related to the age of injection [13] (Fig. 23). Other complications of paraffin injection include migration, inflammatory reaction, infection, sinus tract formation, and tissue necrosis as in other injectable augmentation techniques [6].

### Autologous myocutaneous flap

**Fig. 24:**
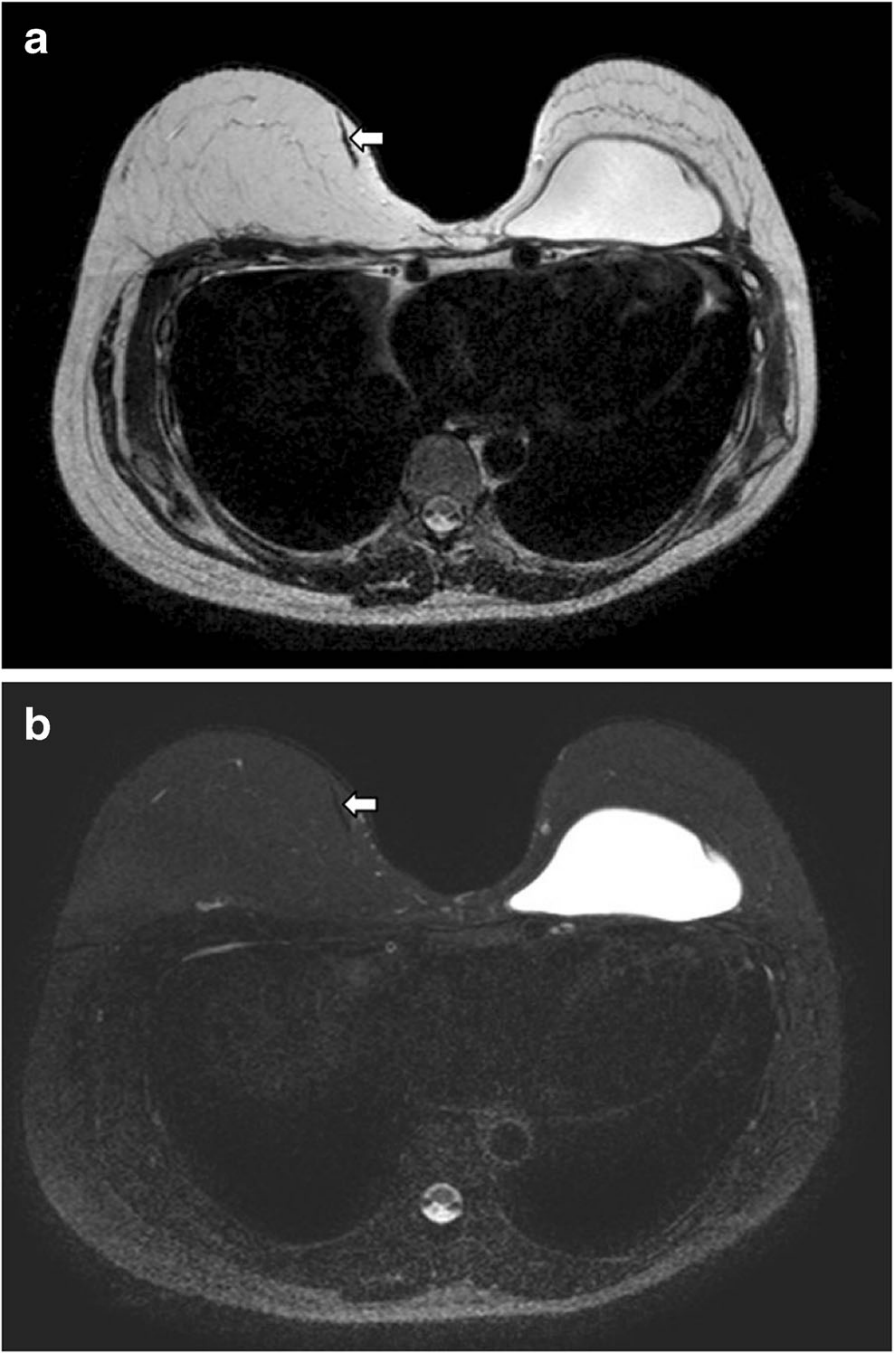
Myocutaneous flaps. These are MR images of a patient with history of bilateral mastectomy and breast reconstruction with TRAM flap at right breast and latissimus dorsi flap plus saline-filled implant at left breast, in order to maintain breasts symmetry. T2-weighted (**a**) and T2-weighted fat-saturated (**b**) axial MR images reveal the complete fatty composition of both breasts without normal glandular tissue, except over the region of saline-filled implant. The denuded dermal layer of the abdominal tissue (arrow) is seen parallel to the skin of right breast

Myocutaneous flap is commonly used in breast reconstruction after surgical removal of breast cancer. The most commonly used flaps are latissimus dorsi and transverse rectus abdominis myocutanaeous (TRAM) flaps (Fig. 24). Latissimus dorsi flap consists of latissimus dorsi muscle and its overlying skin and fat. On MRI, the muscle flap has a tailed appearance at the lateral breast, with the overlying skin and fat flipped and tunnelled from the back to the neobreast.

TRAM flap can be categorized into pedicled flap and free flap, depending on the vascular supply. It consists of the rectus abdominis muscle along with the subcutaneous soft tissue. The atrophied rectus abdominis muscle is located in the centre of the anterior chest wall, in contrast to the eccentric location of the latissimus dorsi flap. On MRI, TRAM flap shows the same signal intensity as fat, with an enhancing contact zone between the flap and the mastectomy site [14].

Fibrosis is frequently seen after radiation therapy. MRI is helpful in differentiating post-operative scarring, post-radiation fibrosis, and tumour recurrence. The former displays low signal intensity on T2-weighted images with no or low level of enhancement [15]. Other common complications include seroma and hematoma in the early postoperative period. Fat necrosis is often seen in the long term, especially for TRAM flap. MRI manifests as a round or irregular mass with central fatty signal, which shows variable contrast enhancement [15].

### Deep inferior epigastric perforator flap

Deep inferior epigastric perforator (DIEP) flap is a recently developed flap technique that preserves the entire rectus abdominis muscle, thus can reduce post-operative pain, recovery time, and risk of abdominal wall weakness and hernias [16]. The normal appearance of DIEP flap is differentiated from a TRAM flap by the absence of atrophied rectus abdominis muscle and its vascular pedicle [16]. Complications of DIEP flaps and their appearances on MRI are similar to TRAM flap, including, fat necrosis, and seroma or hematoma in early post-operative period [16].

### Autologous fat grafting

**Fig. 25:**
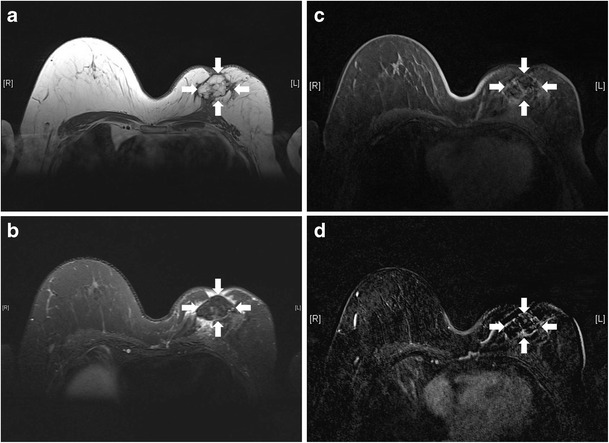
Fat necrosis after autologous fat implant. T1-weighted (**a**), T2-weighted, fat-saturated (**b**), and T1-weighted, fat-saturated (**c**) axial MR images show a multilobulated lesion (arrows) with signal identical to fat in the left breast. Mild perifocal edema is noted (**b**). T1-weighted, post-contrast axial MR image with subtraction (**d**) shows the non-enhancing nature of the fatty masses. A thin rim of enhancement surrounding them is likely due to post-operative changes

Autologous fat grafting is a useful technique in reconstructive breast surgery and is reliable for secondary breast reconstruction [17]. The advantage of autologous fat over synthetic material is the lack of hypersensitivity or foreign-body reaction. It is easy to harvest. Complications of this technique include fat necrosis (Fig. 25), infection, sclerosis, and calcification, causing disfiguration of breasts [6]. MRI is useful in differentiating normal breast fat from fat necrosis as well as early tumour recurrence. It can also assess the viability of the fat graft [18].

### Concurrent breast abnormalities

**Fig. 26:**
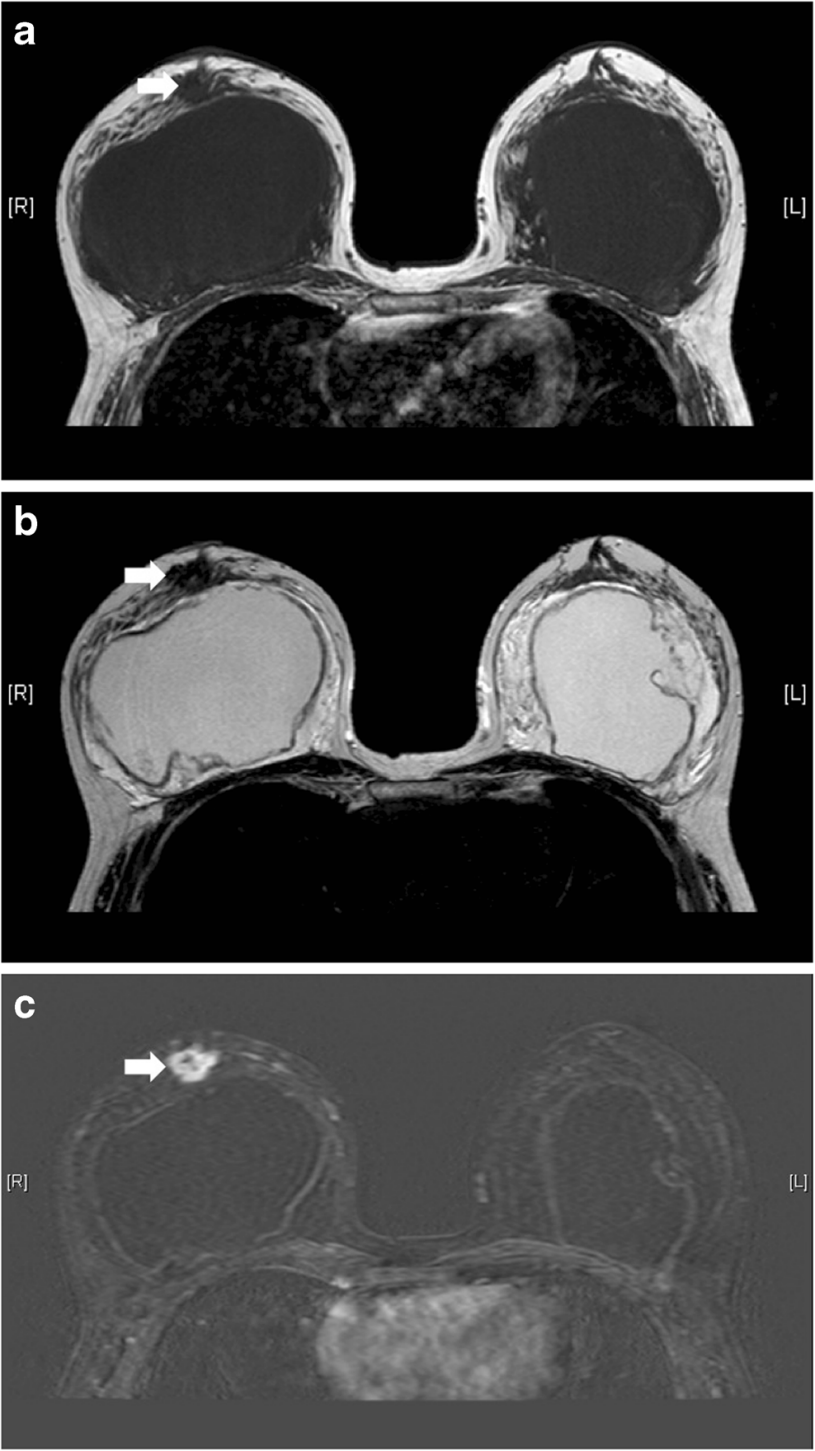
Concurrent breast tumour anterior to right breast implant. T1-weighted (**a**), T2-weighted (**b**), and T1-weighted, post-contrast with subtraction (**c**) axial MR images show a T1 and T2 hypointense lesion with contrast enhancement (arrow) anterior to right breast implant. It was pathologically proven to be invasive ductal carcinoma

Both benign and malignant diseases can also occur in augmented or reconstructed breasts. The most important condition to highlight is breast cancer. Breast augmentation does not increase the risk of breast cancer [19, 20] or cancer at other sites [21]. However, early detection by physical examination or imaging modalities such as mammography may be hindered by the presence of breast implants and post-operative scarring [22] (Fig. 26). Also, there are a number of breast augmentation or reconstruction related conditions that can mimic malignancy as aforementioned. Therefore, radiologists should evaluate the images cautiously, and tissue diagnosis may sometimes be necessary. MRI, particularly with the administration of intravenous contrast, plays an important role in cancer detection as its sensitivity seems not to be reduced by the presence of implants [20].

### Breast implant-associated anaplastic large cell lymphoma

Breast implant-associated anaplastic large cell lymphoma (BIA ALCL) is a newly recognized disease entity, first described by Keech and Creech in 1997 [23], with less than 100 cases reported in the literature [24]. The most common presentations were reported to be peri-implant effusion and palpable mass [25]. Presence of peri-implant effusion beyond 1 year after implant surgery is uncommon and should raise the suspicion of BIA ALCL [24]. Ultrasound and MRI were reported to be the most sensitive imaging modalities to detect effusion [24]. The sensitivity and specificity for detecting the peri-implant fluid by MRI were found to be 82 % and 33 %, respectively, usually associated with thickening and enhancement of the implant capsule, while the sensitivity and specificity of detecting a mass by MRI were found to be 50 % and 95 %, respectively [24].

## Conclusion

There is a wide spectrum of normal and abnormal findings related to breast augmentation and reconstruction. MRI is known to be the imaging tool of choice. Radiologists should be familiar with the imaging appearances to prevent misinterpretation, detect complication, pick up concomitant breast pathologies, as well as facilitate surgical intervention and planning.

## Abbreviations

BIA ALCL: Breast implant-associated anaplastic large cell lymphoma
DIEP flap: Deep inferior epigastric perforator flap
MRI: Magnetic resonance imaging
PAAG: Polyacrylamide gel
TRAM flap: Transverse rectus abdominis myocutanaeous flap
